# Novel Molecular Markers Linked to *Pseudomonas aeruginosa* Epidemic High-Risk Clones

**DOI:** 10.3390/antibiotics10010035

**Published:** 2021-01-01

**Authors:** Wedad Nageeb, Dina H. Amin, Zuhair M. Mohammedsaleh, Rabab R. Makharita

**Affiliations:** 1 Medical Microbiology and Immunology Department, Faculty of Medicine, Suez Canal University, Ismailia 41111, Egypt; 2 Microbiology Department, Faculty of Science, Ain Shams University, Cairo 11566, Egypt; dina.hatem@sci.asu.edu.eg; 3 Department of Medical Laboratory Technology, Faculty of Applied Medical Sciences, University of Tabuk, Tabuk 71491, Saudi Arabia; zsaleh@ut.edu.sa; 4 Botany and Microbiology Department, Faculty of Science, Suez Canal University, Ismailia 41522, Egypt; rabab_makharita@science.suez.edu.eg; 5 Biology Department, Faculty of Science and Arts, Khulais, University of Jeddah, Jeddah 21959, Saudi Arabia

**Keywords:** molecular markers, *Pseudomonas aeruginosa* high-risk clones, population structure, clonal success, MLST, virulence, antibiotic resistance

## Abstract

The population structure of *Pseudomonas aeruginosa* is panmictic-epidemic in nature, with the prevalence of some high-risk clones. These clones are often linked to virulence, antibiotic resistance, and more morbidity. The clonal success of these lineages has been linked to acquisition and spread of mobile genetic elements. The main aim of the study was to explore other molecular markers that explain their global success. A comprehensive set of 528 completely sequenced *P. aeruginosa* genomes was analyzed. The population structure was examined using Multilocus Sequence Typing (MLST). Strain relationships analysis and diversity analysis were performed using the geoBURST Full Minimum Spanning Tree (MST) algorithm and hierarchical clustering. A phylogenetic tree was constructed using the Unweighted Pair Group Method with Arithmetic mean (UPGMA) algorithm. A panel of previously investigated resistance markers were examined for their link to high-risk clones. A novel panel of molecular markers has been identified in relation to risky clones including *arm*R, *amp*R, *nal*C, *nal*D, *mex*Z, mexS, *gyr*AT83I, *gyr*AD87N, *nal*CE153Q, *nal*CS46A, *par*CS87W, *par*CS87L, *amp*RG283E, *amp*RM288R, *pmr*ALeu71Arg, *pmrBGly423Cys*, *nuo*GA890T, *pst*BE89Q, *pho*QY85F, *arn*AA170T, *arn*DG206C, and *gid*BE186A. In addition to mobile genetic elements, chromosomal variants in membrane proteins and efflux pump regulators can play an important role in the success of high-risk clones. Finding risk-associated markers during molecular surveillance necessitates applying more infection-control precautions.

## 1. Introduction

*Pseudomonas aeruginosa* has been declared as one of the “six top priority dangerous microbes” by the infectious disease society of America since 2006 and is still among the list of the most worrying pathogens. According to the European Centre for Disease Control (ECDC) in 2013 and the recent Centers of Disease Control (CDC) antibiotic resistance report in 2019, *P. aeruginosa* was classified as a serious public health threat. It is estimated that 51,000 cases of infection exist each year. It is not considered among the urgent threats but it may worsen and become urgent without ongoing monitoring and prevention activities [1,2]. *P. aeruginosa* is one of the Gram-negative bacteria currently causing serious hospital-acquired infections with very few treatment options [3]. The organism has been classified as one of the six ESKAPE organisms (*Enterococcus faecium*, *Staphylococcus aureus*, *Klebsiella pneumoniae*, *Acinetobacter baumannii*, *Pseudomonas aeruginosa*, and *Enterobacter* species) with emerging clinical importance. This group of organisms has long been known as responsible for the majority of nosocomial infections and they are capable of escaping the action of antimicrobial agents [4]. It is also classified as one of the critical pathogens with an urgent need for new antibiotic development by the World Health Organization (WHO) [5,6].

Sound knowledge of the population structure of a bacterial organism and the molecular markers linked to its high-risk behavior are essential to make meaningful clinical conclusions. This knowledge is important when molecular typing is used in surveillance, epidemic and outbreak investigation, and in the identification of epidemic high-risk clones [7]. The population structure of *Pseudomonas aeruginosa* is consensually believed to be of panmictic-epidemic nature [8], which means that it exhibits a high degree of recombination with random association between loci. Panmixia means random mating and panmictic bacterial populations exhibit the characteristics of linkage equilibrium with high genetic variations at neutral loci [9]. For this type of population structure, better prognostic markers are vital to make better evidence-based patient care decisions [10].

Prognostic markers should have the potential to predict high-risk behavior and should show clear links to disease and patient outcomes. Using such molecular markers will enable directed intervention and will also help in molecular epidemiologic surveillance.

High-risk clones are specific sequence types that have been frequently observed and linked to specific types of behavior including virulence, antibiotic resistance, site specific pathogenicity (e.g., cystic fibrosis, keratitis), or infection outcome. It has been suggested that a limited number of frequently observed widespread clones are responsible for human infections [11], with other clones showing links to higher morbidity and mortality rates in cystic fibrosis patients [12,13,14,15]. Specific dominant multi-drug-resistant (MDR) and extensively drug-resistant (XDR) clones appear to be disseminated in hospitals worldwide. Marked as high-risk clones, they are thought to play a major role in the spread of resistance worldwide [16]. In many studies, these clones accounted for the majority of XDR isolates [17]. *P. aeruginosa* is also considered an important pathogen that determines the biggest morbidity and mortality in cystic fibrosis patients with MDR/XDR strains, being highly linked to disease exacerbations [18].

Some recent reports have identified contaminated bronchoscopes’ rinsing water and connecting tubes as reservoirs for spreading the organism [19]. In addition, previous reports have also identified wastewater networks as trafficking sources between hospital wash basins for pathogenic bacteria including *P. aeruginosa* [20]. This situation makes it important to understand whether the clonal success of epidemic high-risk clones is essentially related to mutational resistance or to horizontally acquired resistance elements, i.e., antibiotic inactivating enzymes or integrons carrying specific gene cassettes. Such an understanding can greatly impact the choice of the best approach required to tackle these high-risk clones and to control their dissemination in hospital environments. High-risk behavior may be encountered as an acquired trait, consequently indicating the higher probability of inter-hospital dissemination of XDR/MDR high-risk clones. The other probability is a conserved lineage-related characteristic which is consistently detected in different hospitals where originally susceptible clones may undergo independent parallel evolution into high-risk clones in different settings by acquiring these markers.

It is proposed that *P. aeruginosa* “high-risk clones” represent distinct lineages highly capable of acquiring and maintaining resistance genes and/or the mobile genetic elements containing these genes when compared with the general *P. aeruginosa* population [21]. However, clonal success in susceptible *P. aeruginosa* high-risk clones tends to be under investigated in most studies, which consequently necessitates the assessment of the genetic markers underlying clonal success in a range of both resistant and susceptible high-risk clones, the point that the current study set has tried to cover.

The primary aim of this study was to conduct an additional investigation of the molecular basis of success of high-risk clones. To achieve that, a set of previously investigated resistance markers including those in quinolone resistance-determining region (QRDR), in efflux pumps operons, in cell membrane-related proteins, and others have been specifically examined. To ensure the diversity and comprehensive representation of the studied set of sequences, phylogenetic analysis and hierarchical clustering of the studied sequences were performed in relation to all known Sequence Types (STs) for *P. aeruginosa*. The analysis performed in this work aims at identifying molecular markers or signatures linked to high-risk clones in *P. aeruginosa*. When such markers are detected, they can be used to efficiently direct infection-control efforts which would consequently reduce the spread of these epidemic clones. For example, a marker linked to high-risk behavior can indicate that the patient or the setting carries high-risk potential and consequently necessitates additional precautionary measures or isolation practices to avoid its transmission.

## 2. Results

The population structure of *P. aeruginosa* was analyzed in a large comprehensive dataset. A large set of public *P. aeruginosa* genomes from the Patric database [22] was studied. The analyzed set included the whole spectrum of resistance profiles for ciprofloxacin, levofloxacin, gentamycin, and amikacin antibiotics. Multilocus sequence typing (MLST) was performed for all sequences according to the previously described typing scheme by Curran [23]. Phylogenetic analysis and hierarchical clustering were performed to evaluate the distribution of the studied set of sequences among all known *P. aeruginosa* genomes. Strain relationships were analyzed using the geoBURST Full Minimum Spanning Tree (MST) algorithm [24], as implemented in the software PHYLOVIZ [25] to construct a minimum spanning tree (MST) of the total set of *P. aeruginosa* strains based on MLST data.

### 2.1. Description of Population Structure and Diversity in the Studied Set of Sequences

Applying geoBURST algorithm at a double locus variant level (DLV) showed 186 clonal complexes with 157 singletons. The most frequent clonal complexes observed were CC 233 consisting of 13 STs (30 sequences), CC17 consisting of 4 STs (13 sequences), CC 395 consisting of 2 STs (14 sequences), CC 316 consisting of 3 STs (10 sequences), and CC 319 consisting of 4 STs (12 sequences). Other examples include CC 446 consisting of 2 STs (10 sequences) and CC 111 consisting of 1 ST (30 sequences). This indicates that the sequences under study probably represent a wide range of diversity based on ST classification. The index of association (I _A_) was calculated to estimate the degree of association and recombination between alleles at different loci based on MLST allelic profile data [26]. When all 528 sequences were analyzed, the standardized index of association (I^S^_A_) = 0.1302 (*p* < 0.001). This indicates linkage equilibrium and low evidence of association among the alleles analyzed. Pairwise variance (V_D_ = 1.4063) was greater than the critical value (L = 0.8007) with the mean genetic diversity of (H) as 0.8648 ± 0.0261. These results support that recombination plays a key role in allele distribution and support the non-clonal structure of the *P. aeruginosa* population based on MLST classification of the studied set of sequences.

The degree of concordance between the two typing schemes used was evaluated using Simpson’s index of diversity (SID: with 95% confidence intervals) and showed that MLST (Simpson’s ID = 0.987 with 95% CI (0.984–0.990)) was more discriminatory than serotyping (Simpson’s ID = 0.856 with 95% CI (0.843–0.869)). Inter-method concordance was also evaluated using the adjusted Wallace coefficient [27]. The adjusted Wallace coefficient shows the probability that two strains classified as the same type by one method will also be classified as the same one when using another method. Adjusted Wallace between ST and serotypes = 0.840 with 95% CI (0.792–0.889), while that between serotypes and STs was significantly low = 0.064 with 95% CI (0.048–0.080), which means that ST can predict serotype with high confidence while the opposite is not true.

**Figure 1:**
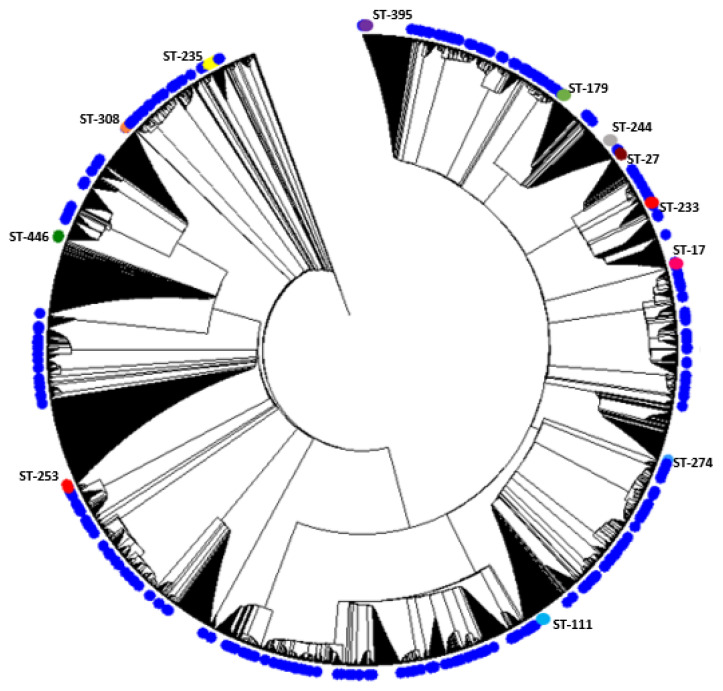
Hierarchical clustering showing the distribution of analyzed sequences among all known Sequence Types STs. Blue circles indicate the position of the studied sequences among all other *P. aeruginosa* sequences. The positions of high-risk clones are also shown on the figure using different colors.

A phylogenetic analysis of the concatenated sequences of the MLST alleles was performed including the study sequences and the entire MLST database. The results of the phylogenetic analysis indicate that the sequences exhibit diversity and show non-clustered distribution among all known STs for the organism. The results are shown in Figure 1. The figure illustrates the position of high-risk clones among all other sets of sequences included in the analysis, which are shown in blue.

### 2.2. Analysis of Different Multilocus Sequence Typing (MLST) Profiles

**Figure 2:**
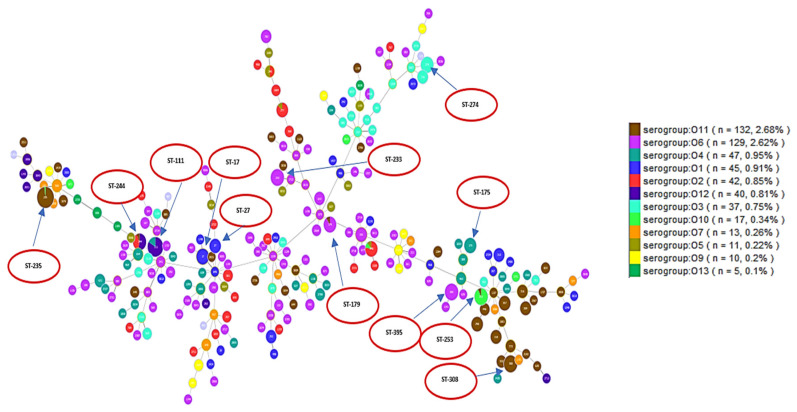
Each serotype is shown in a different color as indicated in the ligand. The figure shows how different serotypes are clustered in relation to different clonal complexes. High-risk clones are shown in red circles.

A population analysis of 528 *P. aeruginosa* sequences was performed and considerable genetic diversity was observed among the MLST results. MLST analysis identified 249 STs among all sequences, including 229 known and 20 novel STs. The international clones ST235 (serotype O11 (98%)) was the most frequently identified in a total of 50 sequences, followed by ST111 (serotype O12 (83.3%) and serotype O4 (16.7%)) which was identified in a total of 30 sequences. ST244 (serotype O12 (53.9%) and serotype O2 (58.5%)) was identified in 13 sequences, while ST308 (serotype O11 (100%)) was identified in 14 sequences. Each of the sequence types of ST395 (serotype O6 (100%)) and ST253 (serotype O10 (92.3%)) were identified in 13 sequences. ST348 (serotype O2 (88.9%)) was identified in nine sequences. ST274 (serotype O3 (100%)) was identified in 10 sequences. ST179 (serotype O6 (90%)) was identified in 10 sequences and ST233 (serotype O6 (100%)) was identified in 13 sequences. ST17 (serotype O1 (100%)) was identified in 10 sequences, while ST27 (serotype O1 (100%)) was identified in six sequences and ST175 (serotype O4 (100%)) in eight sequences. These data are visualized in Figure 2 which shows serotypes overlaid on corresponding STs. Figure 2 shows the minimum spanning tree (MST) analysis of the studied set of *P. aeruginosa* sequences based on MLST data at SLV level. Each circle corresponds to an ST identified in the studied collection of sequences. The area of each circle corresponds to the number of sequences showing a certain ST. The positions of high-risk groups are shown on the graph. In Figure 2, different ST groups are colored based on the corresponding serotype. This gives an idea about the degree of concordance/correlation between the two typing methods.

### 2.3. Resistance Profile of P. aeruginosa Epidemic High-Risk Clones

Among the studied sequences, the international high-risk clone ST235 was the most frequently identified among all STs in the study set (50 sequences). Among its 50 sequences, 13 are ciprofloxacin-resistant, 46 are levofloxacin-resistant, and only 4 sequences are levofloxacin-susceptible. ST111 was the next most frequently observed ST in a total of 30 sequences, with 28 levofloxacin-resistant sequences, 2 levofloxacin-susceptible sequences, 3 ciprofloxacin-resistant sequences, and 1 ciprofloxacin-susceptible sequence. ST244 was identified in 13 sequences; 3 are levofloxacin-susceptible, 10 are levofloxacin-resistant, and 2 are ciprofloxacin-resistant. ST395 was identified in 13 sequences; 8 are levofloxacin-susceptible, 5 are levofloxacin-resistant, and 3 are ciprofloxacin-resistant. ST175 was identified in eight sequences; all are levofloxacin resistant. The cystic fibrosis (CF) clone ST17 was identified in 10 sequences; 4 are levofloxacin-susceptible, 6 are levofloxacin-resistant, and 1 is ciprofloxacin-resistant. Another CF clone, ST274, was identified in 10 sequences; 5 are levofloxacin-susceptible, 5 are levofloxacin-resistant, 1 is ciprofloxacin-resistant, and 1 is ciprofloxacin-susceptible. These results are summarized in Table 1.

For aminoglycosides, 23 sequences with ST235 are amikacin-resistant, 27 are amikacin-susceptible, 3 are gentamycin-resistant, and 10 are gentamycin-susceptible. ST111 included a total of 14 amikacin-resistant sequences, 16 amikacin-susceptible sequences, 3 gentamycin-resistant sequences, and 1 gentamycin-susceptible sequence. Sequences belonging to ST244 included 6 amikacin-resistant sequences, 7 amikacin-susceptible sequences, and 2 gentamycin-resistant sequences. ST395 included 1 amikacin-resistant sequence, 12 amikacin-susceptible sequences, 2 gentamycin-resistant sequences, and 1 gentamycin-susceptible sequence. All sequences with ST175 were amikacin susceptible. Sequences with ST17 included 8 amikacin-susceptible sequences, 2-amikacin resistant sequences, and 1 gentamycin-susceptible sequence. Sequences with ST274 included 9 amikacin-susceptible sequences, 1 amikacin-resistant sequence, 1 gentamycin-resistant sequence, and 1 gentamycin-susceptible sequence (Table 1).

**Figure 3:**
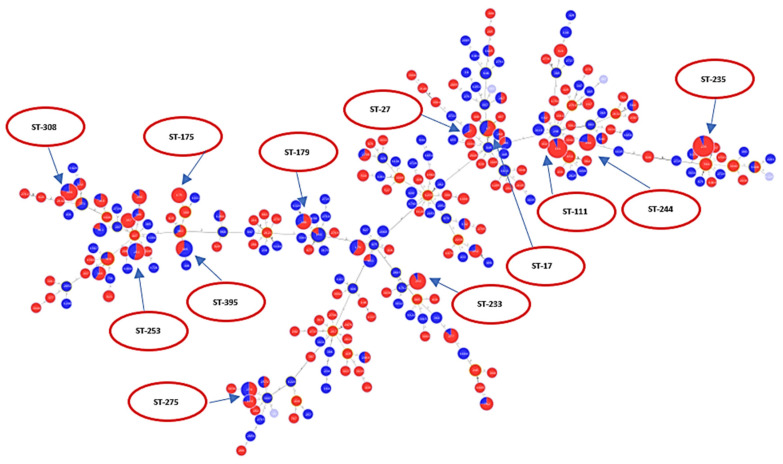
Minimum spanning tree (MST) diagram showing distribution of levofloxacin susceptibility in relation to different STs. Red color: levofloxacin-resistant (*n* = 338); blue color: levofloxacin-susceptible (*n* = 190).

Quinolone and aminoglycoside Minimum Inhibitory Concentration (MIC) values for the high-risk clones observed in the studied collection with their corresponding serotypes are shown in Supplementary Tables S1 and S2. Figures 3, 5, 6, and 8 show the susceptibility of the different studied antibiotic agents in relation to different STs.

### 2.4. Analysis of Molecular Markers in Relation to High-Risk Clones

#### 2.4.1. Levofloxacin-Related Molecular Markers

A total of 528 sequences were analyzed for levofloxacin susceptibility (338 resistant and 190 susceptible). Figure 3 is an MST tree showing levofloxacin susceptibility in relation to different STs. Levofloxacin susceptibility behavior does not show a specific clustering or clonal distribution pattern in relation to the ST classification in general. This figure also shows that ST111, ST235, ST175, and ST233 are majorly composed of resistant sequences, while nearly half of the sequences classified under ST253, ST244, ST17 and ST27 were not resistant. Of those analyzed, a total of 196 sequences belonged to epidemic high-risk clones (ST17 (N = 10), ST27 (N = 6), ST111 (N = 30), ST235 (N = 50), ST175 (N = 8), ST179 (N = 10), ST244 (N = 13), ST233 (N = 13), ST308 (N = 14), ST395 (N = 13), ST532 (N = 3), ST446 (N = 3), ST274 (N = 10), and ST253 (N = 13)).

**Figure 4:**
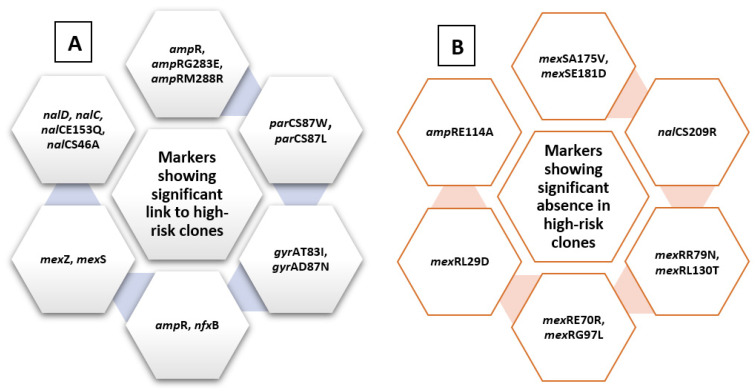
Summary diagram showing levofloxacin-related molecular markers. The figure summarizes markers showing significant presence in high-risk clones (**A**) and those showing significant absence in relation to high-risk clones (**B**).

A chi-square test for independence (with Yates’ continuity correction) indicated significant associations between *amp*R, x^2^ (1, *n* = 528) = 5.7, *p* = 0.017, *phi* = 0.104; *arm* R, x^2^ (1, *n* = 528) = 9.5, *p* = 0.002, *phi* = 0.134; *mexZ*, x^2^ (1, *n* = 528) = 5.9, *p* = 0.015, *phi* = 0.106; *nfx*B, x^2^ (1, *n* = 528) = 6.74, *p* = 0.009, *phi* = 0.113; *mex*S, x^2^ (1, *n* = 528) = 6.2, *p* = 0.013, *phi* = 0.109; *nal*C, x^2^ (1, *n* = 528) = 6.2, *p* = 0.013, *phi* = 0.109; *nal*D, x^2^ (1, *n* = 528) = 3.9, *p* = 0.05, *phi* = 0.085; *gyr*AT83I, x^2^ (1, *n* = 528) = 64.3, *p* < 0.005, *phi* = 0.349; *gyr*AD87N, x^2^ (1, *n* = 528) = 13.23, *p* < 0.005, *phi* = 0.158; *nal*CE153Q, x^2^ (1, *n* = 528) = 10.014, *p* = 0.001, *phi* = 0.139; *nal*CS46A, x^2^ (1, *n* = 528) = 6.23, *p* = 0.013, *phi* = 0.109; *par*CS87W, x^2^ (1, *n* = 528) = 28.03, *p* < 0.005, *phi* = 0.23; *par*CS87L, x^2^ (1, *n* = 528) = 47.6, *p* < 0.005, *phi* = 0.301, *amp*RG283E, x^2^ (1, *n* = 528) = 5.1, *p* = 0.024, *phi* = 0.098; *amp*RM288R, x^2^ (1, *n* = 528) = 7.66, *p* = 0.006, *phi* = 0.121, and high-risk groups. These are listed in Figure 4.

Some markers also showed significant links to specific clones, including *mex*SSer124Arg, *nal*D, *nal*CG71E, *nal*CA186T, *nal*CS46A, *nal*CE153Q, *par*CS87W, *par*CS87L, *gyr*AT83I, *gyr*AD87N, *amp*RG283E, and *amp*RM288R. These are shown in Table 2.

On the other hand, some other markers showed significant absence in relation to high-risk clones, including *mex*SA175V, x^2^ (1, *n* = 528) = 8.5, *p* = 0.004, *phi* = −0.127; *mex*SE181D, x^2^ (1, *n* = 528) = 6.135, *p* = 0.013, *phi* = −0.108; *nal*CS209R, x^2^ (1, *n* = 528) = 18.3, *p* < 0.005, *phi* = −0.186; *mex*RR79N, x^2^ (1, *n* = 528) = 6.7, *p* = 0.01, *phi* = −0.112; *mex*RE70R, x^2^ (1, *n* = 528) = 11.99, *p* = 0.001, *phi* = −0.151; *mex*RL130T, x^2^ (1, *n* = 528) = 8.95, *p* = 0.003, *phi* = −0.13; *mex*RG97L, x^2^ (1, *n* = 528) = 12.5, *p* < 0.005, *phi* = −0.154, *mex*RL29D, x^2^ (1, *n* = 528) = 11.5, *p* < 0.005, *phi* = −0.154, and *amp*RE114A, x^2^ (1, *n* = 528) = 14.32, *p* < 0.005, *phi* = −0.165, summarized in Figure 4.

#### 2.4.2. Ciprofloxacin-Related Molecular Markers

**Figure 5:**
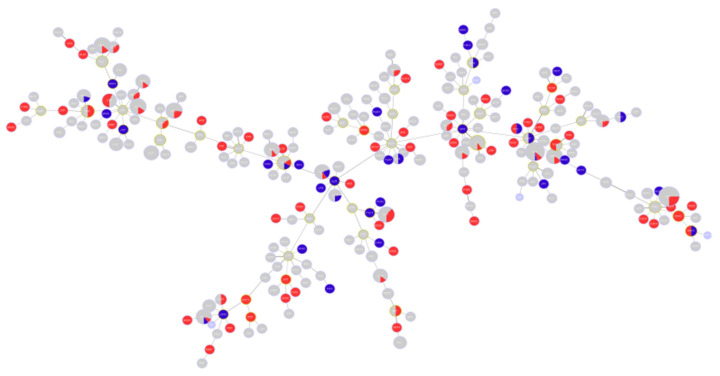
MST diagram showing distribution of ciprofloxacin susceptibility in relation to different STs. Red color: ciprofloxacin-resistant (*n* = 105); blue color: ciprofloxacin-susceptible (*n* = 37); grey color: unknown susceptibility to ciprofloxacin.

A total of 142 sequences were analyzed for ciprofloxacin susceptibility (105 resistant and 37 susceptible). Figure 5 is an MST tree showing ciprofloxacin susceptibility in relation to different STs. The pattern of distribution of antibiotic phenotype in relation to MLST classification is similar to that observed with the levofloxacin MST tree. No clustering of the susceptible phenotype is observed in relation to different STs. This may indicate that information drawn from MLST is not sufficient alone to reflect antibiotic susceptibility behavior in a highly recombining organism like *P. aeruginosa*. Of those analyzed, a total of 39 sequences belonged to epidemic high-risk clones (ST17 (N = 1), ST27 (N = 1), ST111 (N = 4), ST179 (N = 3), ST233 (N = 5), ST235 (N = 12), ST244 (N = 2), ST253 (N = 2), ST274 (N = 2), ST308 (N = 2), ST446 (N = 1), ST395 (N = 3), and ST532 (N = 1)).

A chi-square test for independence (with Yates’ continuity correction) indicated significant associations between *nalC*E153Q, x^2^ (1, *n* = 142) = 7.831, *p* = 0.005, *phi* = 0.235; *parC*S87L, x^2^ (1, *n* = 142) = 6.86, *p* = 0.009, *phi* = 0.22; *gyr*AT83I, x^2^ (1, *n* = 142) = 7.362, *p* = 0.007, *phi* = 0.228; *gyr*AD87N, x^2^ (1, *n* = 142) = 4.668, *p* = 0.031, *phi* = 0.181; *ampR*M288R, x^2^ (1, *n* = 142) = 5.068, *p* = 0.024, *phi* = 0.189, and high-risk groups.

Furthermore, *mexS* Val333Gly, x^2^ (1, *n* = 142) = 10.9, *p* = 0.001, *phi* = 0.277; *nalC*E153Q, x^2^ (1, *n* = 142) = 46.937, *p* < 0.005, *phi* = 0.575; *nalD*I153Q, x^2^ (1, *n* = 142) =4.5, *p* < 0.033, *phi* = 0.179; *gyr*AT83I, x^2^ (1, *n* = 142) = 5.72, *p* = 0.017, *phi* = 0.201; *ampR*G283E, x^2^ (1, *n*= 142) = 15.975, *p* < 0.005, *phi* = 0.335, and *ampR*M288R, x^2^ (1, *n =* 142) =24.26, *p* < 0.005, *phi* = 0.413, showed significant links to ST235.

#### 2.4.3. Amikacin-Related Molecular Markers

**Figure 6:**
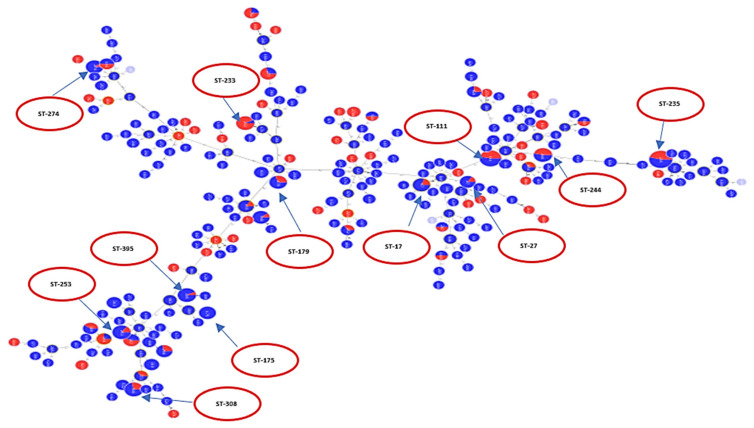
MST diagram showing distribution of amikacin susceptibility in relation to different STs. Red color: amikacin resistant (*n* = 142); blue color: amikacin susceptible (*n* = 386).

A total of 528 sequences were analyzed for amikacin susceptibility (142 resistant and 386 susceptible). Figure 6 is an MST tree showing amikacin susceptibility in relation to different STs. Based on the distribution of high-risk clones seen in Figure 6, amikacin resistance does not appear to correlate with high-risk clones. Except for ST233, the majority of sequences forming all other high-risk STs were mostly susceptible. About half of the isolates forming ST111 and ST235 were resistant. For the ST111 group, 14 sequences were amikacin-resistant and 16 were amikacin-susceptible. For ST235, 23 sequences were amikacin-resistant and 27 were amikacin-susceptible.

Of those analyzed, a total of 196 sequences belonged to epidemic high-risk clones (ST17 (N = 10), ST27 (N = 6), ST111 (N = 30), ST235 (N = 50), ST175 (N = 8), ST179 (N = 10), ST244 (N = 13), ST233 (N = 13), ST308 (N = 14), ST395 (N = 13), ST532 (N = 3), ST446 (N = 3), ST274 (N = 10), and ST253 (N = 13)).

**Figure 7:**
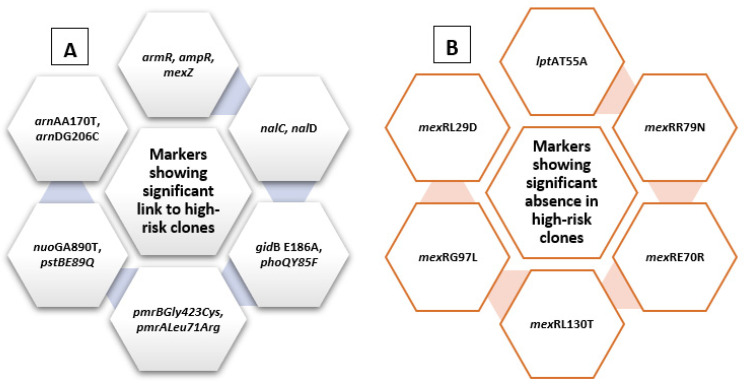
Summary diagram showing amikacin-related molecular markers. The figure summarizes markers showing significant presence in high-risk clones (**A**) and those showing significant absence in relation to high-risk clones (**B**).

A chi-square test for independence (with Yates’ continuity correction) indicated significant associations between *armR* and high-risk groups, x^2^ (1, *n* = 528) = 9.5, *p* = 0.002, *phi* = 0.134. A significant association was also found between each of *nalC*, x^2^ (1, *n* = 528) = 6.218, *p* = 0.013, *phi* = 0.109; *nal*D, x^2^ (1, *n =* 528) = 3.856, *p* = 0.05, *phi* = 0.085; *mexZ*, x^2^ (1, *n* = 528) = 5.904, *p* = 0.015, *phi* = 0.106; *ampR*, x^2^ (1, *n* = 528) = 5.701, *p* = 0.017, *phi* = 0.104; *gid*BE186A, x^2^ (1, *n =* 528) = 89.753, *p* < 0.005, *phi* = 0.412; *pmrBGly423Cys*, x^2^ (1, *n* = 528) = 21.478, *p* < 0.005, *phi* = 0.202; *pmrALeu71Arg*, x^2^ (1, *n* = 528) = 14.546, *p* < 0.005, *phi* = 0.166; *nuo*GA890T, x^2^ (1, *n* = 528) = 16.236, *p* < 0.005, *phi* = 0.175; *pstBE89Q*, x^2^ (1, *n =* 528) = 6.92, *p* = 0.009, *phi* = 0.114; *arn*AA170T, x^2^ (1*, n =* 528) = 3.878, *p* = 0.049, *phi* = 0.086; *arn*DG206C, x^2^ (1, *n* = 528) = 3.878, *p* = 0.049, *phi* = 0.086; and *phoQY85F*, x^2^ (1, *n* = 528) = 60.031, *p* < 0.005, *phi* = 0.337, and high-risk groups. These are summarized in Figure 7.

Some markers appeared to be conserved to specific high-risk clones, showing very high effect sizes, including *phoQY85F, nuo*GA890T, *pstBE89Q, arn*AA170T, *gid*BE186A, and *arn*DG206C. Table 3 summarizes amikacin molecular markers showing links to specific high-risk clones.

*phoQY85F* showed high conservation to ST235 (x^2^ (1, *n* = 528) = 400.38, *p* < 0.005), with high effect size (*phi* = 0.871). The marker was identified in 48/50 ST235 sequences and rarely identified in other high-risk clones. *nuo*GA890T was exclusively identified in all sequences with ST395 (13/13) and not identified at all in any other high-risk clone (x^2^ (1, *n* = 528) = 455.82, *p* < 0.005), showing very high effect size (*phi* = 0.929). Similarly, *pstBE89Q* showed high conservation to ST233. It was identified in all sequences with ST233 (13/13) and not identified at all in any other high-risk clone assessed (x^2^ (1, *n* = 528) = 338.5, *p* < 0.005, *phi* = 0.801). Similarly, both *arn*AA170T and *arn*DG206C were exclusively identified in ST233 (13/13 sequences) and not in any other high-risk clone among those assessed; x^2^ (1, *n* = 528) = 292.64, *p* < 0.005, *phi* = 0.744, and x^2^ (1, *n* = 528) = 292.64, *p* < 0.005, *phi* = 0.744, respectively. On the other hand, *gid*B E186A showed significant conservation in ST235 (49/50 sequences); x^2^ (1, *n* = 528) = 493.946, *p* < 0.005, with very high effect size, *phi* = 0.967.

On the other hand, some other markers showed absence in relation to high-risk clones, including each of *lpt*AT55A, x^2^ (1, *n* = 528) = 22.8, *p* < 0.005, *phi* = −0.208; *mex*RR79N, x^2^ (1, *n* = 528) = 6.669, *p* = 0.01, *phi* = −0.112; *mex*RE70R, x^2^ (1, *n* = 528) = 11.995, *p* = 0.001, *phi* = −0.151; *mex*RL130T, x^2^ (1, *n* = 528) = 8.953, *p* = 0.003, *phi* = −0.130; *mex*RG97L, x^2^ (1, *n* = 528) = 12.523, *p* < 0.005, *phi* = −0.154, and *mex*RL29D, x^2^ (1, *n* = 691) = 12.502, *p* < 0.005, *phi* = −0.154 (Figure 7).

#### 2.4.4. Gentamycin-Related Molecular Markers

**Figure 8:**
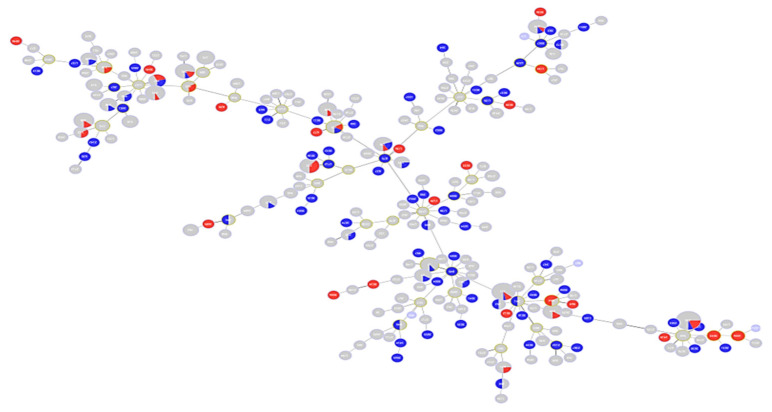
MST diagram showing distribution of gentamycin susceptibility in relation to different STs. Red color: gentamycin resistant (*n* = 57); blue color: gentamycin susceptible (*n* = 82); grey color: unknown susceptibility to gentamycin.

A total of 139 sequences were analyzed for gentamycin susceptibility phenotype (57 resistant and 82 susceptible). Figure 8 is an MST tree showing gentamycin susceptibility in relation to different STs. The distribution of gentamycin susceptibility and resistance in Figure 8 shows that both gentamycin-resistant and gentamycin-susceptible isolates are evenly distributed all over the MST tree. This may indicate that the antibiotic susceptibility phenotype relates to different STs at different distances and is not clustering in clones or in relation to specific clonal complexes. This is also similar to what was observed with quinolones in Figure 3 and Figure 5. Furthermore, the high-risk clones shown in the graph include both resistant and susceptible sequences. Of those analyzed, a total of 39 sequences belonged to epidemic high-risk clones (ST523 (N = 1), ST446 (N = 1), ST395 (N = 3), ST308 (N = 2), ST274 (N = 2), ST253 (N = 1), ST244 (N = 2), ST235 (N = 13), ST233 (N = 5), ST179 (N = 3), ST111 (N = 4), ST27 (N = 1), and ST17 (N = 1)).

A chi-square test for independence (with Yates’ continuity correction) indicated significant association between *nalC*E153Q and high-risk groups; x^2^ (1, *n* = 139) = 22.475, *p* < 0.005, *phi* = 0.402. Significant associations were also found between each of *gidB*E97Q, x^2^ (1, *n* = 139) = 36.772, *p* < 0.005, *phi* = 0.514; *gidB*E186A, x^2^ (1, *n* = 139) = 36.772, *p* < 0.005, *phi* = 0.514; *pst*BE89Q, x^2^ (1, *n* = 139) = 4.989, *p* = 0.026, *phi* = 0.189; and *arn*DG206C, x^2^ (1, *n* = 139) = 4.989, *p* = 0.026, *phi* = 0.189 and high-risk groups. Gentamycin molecular markers showing significant links to specific high-risk clones are summarized in Table 4.

## 3. Discussion

Understanding the reason for the success of epidemic high-risk clones is essential for designing treatment and infection control strategies [28]. The specific genetic resistance markers of these high-risk clones were described in detail for the first time by Cabot et al. [29]. These include multiple combinations of chromosomal mutations and/or horizontally acquired resistance elements. The mosaic nature observed for both chromosomal and acquired resistance elements in relation to high-risk clones necessitates being cautious before making conclusions about the molecular bases of success for these clones [30]. Correa et al. (2015) showed that the dissemination of extensively drug-resistant *P. aeruginosa* has been repeatedly linked to the presence of mobile genetic elements that would facilitate their successful spread and clonal dissemination [21]. Gene cassettes carried by class 1 integrons has also been suggested as an underlying cause for clonal success [31,32].

The analysis performed in this study used previously investigated quinolone and aminoglycoside resistance markers to study their correlation with high-risk clones. The identified high-risk related molecular markers for the studied quinolone group included *amp*R*, mex*Z, *arm*R, *nfx*B, *mex*S, *nal*C, *nal*D, *gyr*AT83I, *gyr*AD87N, *nal*CE153Q, *nal*CS46A, *par*CS87W, *par*CS87L, *amp*RG283E, and *amp*RM288R.

Interestingly, it was observed that 41 sequences out of 50 within the high-risk group ST235 showed the following cluster/combination of molecular markers: *mex*Z, *arm*R, *nfx*B, *mex*S, *mex*R, and *nal*C. *gyr*AT83I was identified in 36/50 sequences and *nal*CS46A in 43/50 sequences. Both *gyr*AT83I and *par*CS87L showed significant links to high-risk clones and specifically to ST235 and ST111, exhibiting the highest effect sizes for individual mutations among all those tested.

Similarly observed for ST111, 27 sequences out of 30 showed the same cluster of molecular markers (*mex*Z, *arm*R, *nfx*B, *mex*S, *mex*R, and *nal*C). *gyr*AT83I was identified in 23/30 sequences and *nal*CS46A was identified in 24/30 sequences. Both showed statistically significant associations.

The findings from the current study can be supported by findings from the literature showing *mex*Z G195E, leading to MexXY overexpression, and *gyr*AT83I among the variants observed in relation to the spread of ST175 XDR phenotype. These were also identified by Kos et al. [3] among mutational resistance mechanisms showing frequent occurrence in both ST111 and ST235 high-risk clones [3]. Other findings by Treepong et al. (2017) also support the link between each of *gyr*AT83I and *par*CS87(80)I and the clone ST235 [33] A recent study has also identified a wide range of mutations in all efflux pump regulators in relation to high-risk clones including *nal*CE153Q, thus supporting findings from the current analysis [34].

Other recent observations also support the same findings, demonstrating the importance of QRDR-related mutations in high-risk clones [35]. In the same study, Horna et al. (2019) identified QRDR mutations in all sequences belonging to ST235 and ST357.

The molecular markers identified for the aminoglycoside group in relation to high-risk clones from the current analysis included *arm*R, *amp*R, *nal*C, *nal*D, *mex*Z, *pmr*ALeu71Arg, *pmr*BGly423Cys*, nuo*GA890T, *pst*BE89Q, *pho*QY85F, *arn*AA170T, *arn*DG206C, and *gid*BE186A. The pattern of their distribution seemed to be highly conserved for specific markers in relation to specific high-risk clones. Markers showing high conservation to specific high-risk clones with very high effect sizes included *phoQY85F* and *gid*B E186A with ST235, *nuo*GA890T with ST395, and *pstBE89Q, arn*AA170T, and *arn*DG206C with ST233.

Although it was shown by Chowdhury et al. [31] that the presence of resistance gene cassettes carried by class 1 integrons may be a characteristic of ST235 clonal lineage, there is no sufficient evidence to show whether this link is related to MDR behavior or whether it is an inherent character of the ST235 lineage. *nfx*B gene, the *pho*Q variant F76Y, and the *pmr*B variant V15I were all identified in all ST235 sequences evaluated from the same study [31]. These findings support the findings from the current analysis about the possible important link of *nfx*B, *pho*Q, and *pmr*AB variants to the ST235 clone.

Findings from the current analysis may also be supported by other recent findings shown by Pelegrin et al. (2019) [34], who identified the same variant *pho*QY85F as highly conserved in ST235. The same study has also shown that *pmr*ALeu71Arg was frequently identified in both ST235 and ST446. Multiple other variants have also been identified in *nuo*G in relation to high-risk clones from the same study [34]. On the other hand, both *arn*AA170T and *arn*DG206C, previously identified as resistance markers to aminoglycosides (under preparation for publication), have also shown significant links to high-risk behavior, especially with ST233, showing very high effect size.

Changes in PhoP-PhoQ activity have been implicated in resistance to cationic antimicrobial peptides as a result of lipopolysaccharide (LPS) modification [36,37,38]. Similar changes may also underlie high-risk behavior, and this could explain the current findings.

*pmr*ALeu71Arg appeared to be highly conserved in some high-risk clones including ST308 (12/14 sequences), ST233 (12/13 sequences), ST253 (11/13 sequences), ST179 (10/10 sequences), and ST175 (8/8 sequences). These findings are supported by findings from a recent study showing upregulation of *pmr*AB and *pho*PQ in relation to high-risk clones and colistin resistance [39]. In addition, some mutations in the two-component sensor-regulator system *pmr*AB have been linked to a changed aminoglycoside resistance phenotype. These include *pmr*ALeu71Arg located within the signal receiver domain of the response regulator. While activation of each of the two systems separately only showed slight increases in MIC, combined activation led to a four-fold increase in the tobramycin MIC [40].

These findings collectively support findings from the literature showing that mutations in several two-component regulatory systems including *pmr*AB, *pho*PQ, and the related overexpression of the *arn*BCADTEF-*pmr*E operon can lead to lipid A modification. This effect proved to be associated with polymyxin resistance in *P. aeruginosa* [41]. A similar effect may also lead to aminoglycoside resistance or high-risk behavior, which may explain the current findings.

## 4. Materials and Methods

As a primary step to explore the specific molecular markers explaining the global success of epidemic high-risk clones, the population structure of *P. aeruginosa* in a large comprehensive dataset was analyzed. A large set of public *P. aeruginosa* genomes from the Patric database [22] were studied. The studied sequences are referred to in Supplementary Table S3. The analyzed set included the whole spectrum of resistance profiles for ciprofloxacin, levofloxacin, gentamycin, and amikacin antibiotics. An extensive panel of molecular markers studied and identified in a previous work in relation to antibiotic resistance (data not shown here) were evaluated in this study for their potential relation to clonal success.

### 4.1. Multilocus Sequence Typing (MLST) and Serotypes (O-type) Analysis

MLST was performed for all sequences according to the previously described typing scheme by Curran [23]. An ST was assigned to each unique allelic profile according to the *P. aeruginosa* PubMLST database (http://pubmlst.org/paeruginosa/). Whole-genome sequence data (WGS) for the selected sequences from Patric database (528 genomes) were used to identify STs using the method publicly available at www.cbs.dtu.dk/services/MLST [42]. WGS data were also used to determine the serogroups of all studied isolates based on the sequence of O-specific antigen (OSA) gene cluster using the *P. aeruginosa* serotyper (PAst) web tool available on the Center for Genomic Epidemiology (CGE) service platform (https://cge.cbs.dtu.dk/services/PAst-1.0/) [43].

### 4.2. Phylogenetic Analysis

Phylogenetic analysis and hierarchical clustering were performed to evaluate the distribution of the studied set of sequences among all known *P. aeruginosa* genomes. The genes encoding the metabolic enzymes, *acs*A, *aro*E, *gua*A, *mut*L, *nuo*D, *pps*A, and *trp*E, which are commonly used for MLST typing, were used. Concatenated sequences of these genes for the studied set of 528 genomes, as extracted from the MLST output tool provided by https://cge.cbs.dtu.dk/services/MLST/, and concatenated sequences of all other known STs for *P. aeruginosa*, as extracted from the *Pseudomonas aeruginosa* MLST website, available at https://pubmlst.org/paeruginosa/, were aligned using the MUSCLE option [44] implemented in the software MEGA 7 [45]. The phylogenetic tree of the concatenated genes was constructed using the UPGMA algorithm.

### 4.3. Population Structure and Diversity Analysis

Strain relationships were analyzed using the geoBURST Full MST algorithm [24], as implemented in the software PHYLOVIZ [25], to construct a minimum spanning tree (MST) of the total set of *P. aeruginosa* strains based on MLST data according to the steps shown in Phyloviz documentation release 2.0, available at http://www.phyloviz.net/. Clonal complexes (CCs) were defined in the current analysis as complexes or “groups of studied sequences” containing at least three STs sharing the same allele numbers in at least five of seven loci. Isolate-specific metadata, including serotypes, quinolone, and aminoglycoside resistance data, were then overlaid on top of the minimum spanning tree. Allelic linkage disequilibrium was assessed with two test options of both Monte Carlo methods and Parametric with 100 resampling using LIAN version 3.7 [26], available at http://guanine.evolbio.mpg.de/cgi-bin/lian/lian.cgi.pl/query.

The standardized index of association (I^S^_A_) and the mean genetic diversity (H) were used to assess the linkage equilibrium and degree of association between alleles [46,47].

Discriminatory power (Simpson’s index) and concordance (cluster agreement) between 2 typing methods (adjusted Rand index and adjusted Wallace) were evaluated according to Carriço et al. [48] using the web source http://www.comparingpartitions.info/.

The degree of concordance between the two typing schemes used was first evaluated. Simpson’s index of diversity (SID: with 95% confidence intervals) was used as described by Hunter and Gaston [49]. Inter-method concordance was also evaluated using the adjusted Wallace coefficient [27].

### 4.4. Resistance Genes and Markers Correlations

A primary literature review to extract genes and gene variants associated with quinolone and aminoglycoside resistance was carried out on PMC PubMed Central, ACADEMIC SEARCH COMPLETE (EBSCO host), and ScienceDirect using the following search criteria:

“*Pseudomonas aeruginosa*” [title/abstract] AND “aminoglycosides resistance”[title/abstract]

“*Pseudomonas aeruginosa*” [title/abstract] AND “Quinolone resistance”[title/abstract].

Secondary more specific searches were also conducted using search criteria “Efflux pumps OR Target mutations AND *Pseudomonas aeruginosa*”. All search results were analyzed to extract variants and genes with function related to antibiotic resistance for the studied antibiotic groups. A large set of data was generated. The correlations of these markers were further tested in relation to antibiotic susceptibility and were prioritized based on their diagnostic predictive values (data not shown here).

High-risk clones studied here included the most well-known and well-studied groups as extracted by Oliver et al. (2015) [11] and relevant review articles. The final set of the previously identified and prioritized antibiotic-resistance-related markers were then explored for their correlation with the well-studied *P. aeruginosa* high-risk STs in order to study their potential to explain the success of epidemic high-risk clones. Markers that have been previously identified from the literature in relation to antibiotic resistance and those novel ones are listed in Table 5. Chi-square test for independence (with Yates’ continuity correction) was used to compare groups. A *p*-value of < 0.05 was considered as statistically significant.

The panel of previously tested ciprofloxacin and levofloxacin resistance molecular markers was analyzed for possible correlation with high-risk clones. These markers included *mex*Z, *nal*CS46A, *nal*CS209R, *nal*CG71E, *gyr*AT83I, *gyr*AD87N, *par*CS87W, *par*CS87L, *nal*CE153Q, *nal*CThr50pro, *mex*S gene, *nal*D gene, *nfx*B gene, *arm*R gene, *par*EV460G, *amp*RD135N, *amp*RG283E, and *amp*RM288R.

For gentamycin and amikacin, the molecular markers tested included *pho*QY85F, *nuo*GA890T, *pst*BE89Q, *lpt*AT55A, *lptA*R62S, *fao*AT385A, *arn*AA170T, *arn*DG206C, *mex*RR79N, *mex*RE70R, *mex*RL130T, *mex*RG97L, *mex*RL29D, *mex*Z, *amp*R gene, *pmr*BGly423Cys, *pmr*ALeu71Arg, *fus*A1D588G, *gid*BE186A, *arm*R (PA3719), *nal*C gene, *nal*D, *nal*Dser32Asn, *gid*BQ28K, *gid*BE97Q, *nal*CE153Q, and *amp*RA51T.

## 5. Conclusions

The findings from the current study point to the importance of membrane protein variants and efflux pump regulators in the success of high-risk clones. The functional modifications caused by these variants may give the clones their success in addition to being related to resistance or virulence characteristics. Although inactivating enzymes were not assessed in the current study, a new group of mutational variants in chromosomal genes related to efflux pumps, efflux pump regulators, and membrane proteins showing strong correlations in a large diverse set of sequences can support the assumption that horizontally acquired elements, whether through plasmids or integrons, are not the sole underlying molecular elements behind the success and spread of epidemic high-risk clones. Variants identified from the current analysis can represent biologic markers showing increased fitness and leading to the acquisition of specific adaptive or beneficial traits. These variants may also represent an adaptation to chronic infections. However, this needs to be further investigated by studying the variants’ biologic effect, which is beyond the objectives of this work.

*P. aeruginosa* sub-lineages have been previously observed to show independent signatures of adaptation that may result into distinct biologic activities [50] and this support the assumption from the current study. Although not much information is available on the mutation rate of high-risk *P. aeruginosa* clones, it has been suggested that the mutator phenotypes observed in high-risk clones may play a role in the adaptability required for the global success and dissemination of high-risk clones showing markers similar to those observed in chronic infections [51,52,53]. This assumption can also be supported by findings from the current analysis. Further evidence suggesting the independent acquisition of adaptive characters is in the findings of mutational resistance arising independently across distinct phylogenetic lineages and contributing to the mutation-driven evolution of the *P. aeruginosa* population structure [54].

**Figure 9:**
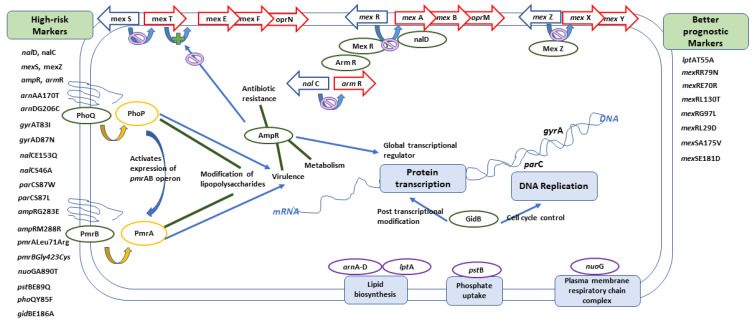
A graphical summary showing the role of different genes within the cell.

In conclusion, *arm*R, *amp*R, *nal*C, *nal*D, *mex*Z, mexS, *gyr*AT83I, *gyr*AD87N, *nal*CE153Q, *nal*CS46A, *par*CS87W, *par*CS87L, *amp*RG283E *amp*RM288R, *pmr*ALeu71Arg, *pmrBGly423Cys, nuo*GA890T, *pst*BE89Q, *pho*QY85F, *arn*AA170T, *arn*DG206C, and *gid*BE186A can all be considered as molecular markers of high-risk behavior, while other markers including *lpt*AT55A, *mex*RR79N, *mex*RE70R, *mex*RL130T, *mex*RL29D, *mex*SA175V, *mex*SE181D, and *mex*RG97L can indicate the absence of risky clones and, consequently, better prognosis. Figure 9 shows a graphical summary of the main conclusions, representing the role of different genes within the cell. Finding risk-associated markers during hospital molecular surveillance or outbreak investigation necessitates applying more infection-control precautions to prevent transmission of such risky clones.

## Figures and Tables

**Table 1:** Summary of sequences belonging to high-risk clones with their corresponding quinolone and aminoglycoside susceptibility.

**High-risk clone**	**Number of sequences**	**Ciprofloxacin-resistant**	**Ciprofloxacin-susceptible**	**Unrecorded ciprofloxacin-susceptibility**	**Levofloxacin-resistant**	**Levofloxacin-susceptible**	**Gentamycin-resistant**	**Gentamycin-susceptible**	**Unrecorded gentamycin-susceptibility**	**Amikacin-resistant**	**Amikacin-susceptible**
ST235	50	13	-	37	46	4	3	10	37	23	27
ST111	30	3	1	26	28	2	3	1	26	14	16
ST244	13	2	-	11	10	3	2	-	11	6	7
ST395	13	3	-	10	5	8	2	1	10	1	12
ST175	8	-	-	8	8	-	-	-	8	-	8
ST17	10	1	-	9	6	4	-	1	9	2	8
ST274	10	1	1	8	5	5	1	1	8	1	9

(-) sign refers to (No sequences) in the category specified.

**Table 2:** Levofloxacin molecular markers showing significant association with specific high-risk clones.

Molecular Marker	High-Risk Clone	Chi-Square	*p*-Value	Phi Coefficient
*mex*SSer124Arg	ST235	39.6	<0.005	0.274
*mex*SSer124Arg	ST395	14.06	<0.005	0.163
*nal*D	ST395	3.97	0.046	0.274
*nal*CG71E	ST395	5.7	0.017	0.103
*nal*CA186T	ST233	147.6	<0.005	0.529
*nal*CS46A	ST235	3.06	0.08	0.076
*nal*CS46A	ST244, ST253	4.44	0.035	0.092
*nal*CE153Q	ST235	71.06	<0.005	0.367
*nal*ID153Q	ST235	20.7	<0.005	0.198
*par*CS87W	ST274	7.9	0.005	0.122
*par*CS87L	ST111	17.09	<0.005	0.18
*par*CS87L	ST233	32.3	<0.005	0.248
*par*CS87L	ST235	49	<0.005	0.306
*par*CS87L	ST244	9.5	0.005	0.135
*par*CS87L	ST308	16.6	<0.005	0.178
*gyr*AT83I	ST111	25.7	<0.005	0.22
*gyr*AT83I	ST233	20.1	<0.005	0.195
*gyr*AT83I	ST235	35.42	<0.005	0.259
*gyr*AT83I	ST244	4.5	0.035	0.092
*gyr*AT83I	ST308	12.7	<0.005	0.155
*gyr*AD87N	ST395	7.13	0.008	0.116
*amp*RG283E	ST235	49.85	<0.005	0.307
*amp*RG283E	ST308	9.78	0.002	0.136
*amp*RM288R	ST235	82.776	<0.005	0.396
*amp*RM288R	ST308	18.658	<0.005	0.188

**Table 3:** Amikacin molecular markers showing significant associations with specific high-risk clones.

Molecular Marker	High-Risk Clone	Chi-Square	*p*-Value	Phi Coefficient
*phoQY85F*	ST235	400.38	<0.005	0.871
*nuo*GA890T	ST395	455.82	<0.005	0.929
*pstBE89Q*	ST233	338.5	<0.005	0.801
*arn*AA170T	ST233	292.64	<0.005	0.744
*arn*DG206C	ST233	292.64	<0.005	0.744
*gid*BE186A	ST235	493.946	<0.005	0.967
*lpt*AR62S	ST446	85.48	<0.005	0.402
*fao*AT385A	ST244	121.92	<0.005	0.481
*pmrBGly423Cys*	ST111	22.492	<0.005	0.206
*pmrBGly423Cys*	ST235	28.103	<0.005	0.231
*pmrBGly423Cys*	ST253	14.338	<0.005	0.165
*pmrBGly423Cys*	ST308	12.493	<0.005	0.154
*pmrALeu71Arg*	ST17	29.38	<0.005	0.236
*pmrALeu71Arg*	ST233	30.868	<0.005	0.242
*pmrALeu71Arg*	ST253	24.145	<0.005	0.214
*pmrALeu71Arg*	ST308	27.034	<0.005	0.226
*pmrALeu71Arg*	ST175	23.414	<0.005	0.211
*pmrALeu71Arg*	ST179	29.38	<0.005	0.236
*pmrALeu71Arg*	ST532	8.696	0.003	0.128

**Table 4:** Gentamycin molecular markers showing significant associations with specific high-risk clones.

Molecular Marker	High-Risk Clone	Chi-Square	*p*-Value	Phi Coefficient
*nal*DSer32Asn	ST235	3.955	0.047	0.169
*nalC*E153Q	*ST235*	98.99	<0.005	0.844
*pmr*ALeu71Arg	ST308	5.594	0.018	0.201
*pmr*ALeu71Arg	ST233	14.298	<0.005	0.321
*pmr*ALeu71Arg	ST179	8.453	0.004	0.247
*pst*BE89Q	ST233	84.93	<0.005	0.782
*arn*A A170T	ST233	74.917	<0.005	0.734
*arn*DG206C	ST233	84.93	<0.005	0.782
*gidB*E97Q	ST235	139	<0.005	1
*gidB*E186A	ST235	139	<0.005	1

**Table 5:** Markers tested for their correlation with high-risk clones.

Molecular Markers Previously Identified in Relation to Antibiotic Resistance	Newly Identified Molecular Markers
*mex*Z *nal*CS46A *nal*CS209R *nal*CG71E *gyr*AT83I *gyr*AD87N *par*CS87W *par*CS87L *nal*CE153Q *nal*CThr50pro *mex*S gene *mex*SA175V *mex*SE181D *nal*D gene *nfx*B *arm*R *par*EV460G *amp*RD135N *nal*C *amp*R *pmr*BGly423Cys *pmr*ALeu71Arg *nal*Dser32Asn *amp*RA51T *amp*RG283E *amp*RM288R	*pho*QY85F *nuo*GA890T *pst*BE89Q *lpt*AT55A *lpt*AR62S *fao*AT385A *arn*AA170T *arn*DG206C *mex*RR79N *mex*RE70R *mex*RL130T *mex*RG97L *mex*RL29D *fus*A1D588G *gid*BE186A *gid*BQ28K *gid*BE97Q

## Data Availability

Primary sequence data analyzed in this study are available in Supplementary Materials S1, S2, and S3.

## Supplementary Materials

The following are available online at https://www.mdpi.com/2079-6382/10/1/35/s1, Table S1: Quinolones’ MIC values for the studied set of sequences and their corresponding STs and serotypes; Table S2: Aminoglycosides’ MIC values for the studied set of sequences and their corresponding STs and serotypes; Table S3: ID of studied set of sequences from the Patric database and their accession numbers.
